# *Notes from the Field:* Follow-Up on 11 Infants Born to Women with Evidence of Zika Virus Infection During Pregnancy — Los Angeles County, 2016

**DOI:** 10.15585/mmwr.mm6749a5

**Published:** 2018-12-14

**Authors:** Curtis Croker, Amy Marutani, Marita Santos, Susan Hathaway, Bessie Hwang

**Affiliations:** 1 Acute Communicable Disease Control Program, Los Angeles County Department of Public Health, Los Angeles, California.

Microcephaly and other birth defects have been identified among infants born to women with Zika virus infection during pregnancy (*1*–*4*). In accordance with CDC recommendations (*5*), the Los Angeles County (LAC) Department of Public Health implemented surveillance to assess the health of infants born to women with evidence of Zika virus infection during pregnancy at birth and at ages 2, 6, and 12 months. These recommendations included testing all such infants at birth for Zika virus.

During 2016, 11 infants were born to women in LAC who met the Council of State and Territorial Epidemiologists case definition (*6*) for confirmed (four infants) or probable (seven infants) Zika virus infection (Table). Follow-up through age 12 months was completed for nine infants; two infants (numbers 3 and 5) were evaluated at birth, and their parents declined to participate after delivery. All infants appeared healthy and normal at the last available assessment, with normal head circumference measurements. Zika virus immunoglobulin M (IgM) testing of serum was completed on eight infants at birth; all test results were negative. Three of these eight infants were also tested for Zika virus RNA in urine and in serum; all test results were negative.

**TABLE:** Follow-up* of 11 infants born to women with Zika virus infection during pregnancy — Los Angeles County, 2016

Maternal, fetal, perinatal, and infant testing	Infant no.										
	1	2	3	4	5	6	7	8	9	10	11
**Mother**											
Symptomatic	yes	yes	no	no	no	yes	yes	yes	yes	no	no
Zika IgM and PRNT^†^	positive	positive	positive	positive	positive	positive	positive	positive	positive	positive	positive
Zika RNA by PCR^§^	—	—	—	—	—	negative	—	negative	—	—	—
Dengue IgM and PRNT^¶^	negative	positive	negative	negative	positive	positive	positive	positive	negative	positive	positive
Case status	conf.	prob.	conf.	conf.	prob.	prob.	prob.	prob.	conf.	prob.	prob.
**Fetus**											
Cranial ultrasound	negative	negative	negative	positive	negative	negative	negative	negative	negative	NT	negative
Amniotic fluid (Zika RNA)	NT	NT	NT	negative	NT	NT	negative	NT	NT	NT	NT
**Zika RNA in tissue**											
Central placenta	negative	negative	NT	negative	negative	NT	negative	negative	negative	NT	negative
Placental membrane	negative	negative	NT	negative	negative	NT	negative	NT	negative	NT	negative
Umbilical cord membrane	positive	negative	NT	negative	NT	NT	negative	NT	negative	NT	negative
**Infant**	negative	NT	NT	negative	negative	negative	negative	negative	negative	NT	—
Zika IgM (serum)**	negative	NT	NT	negative	negative	negative	negative	negative	negative	NT	negative
Zika RNA by PCR (urine)**	NT	NT	NT	negative	NT	negative	NT	negative	NT	NT	NT
Zika RNA by PCR (serum)**	NT	NT	NT	negative	NT	negative	NT	negative	NT	NT	NT
Apgar score (5 minutes)	9/10	9/10	9/10	9/10	9/10	9/10	9/10	9/10	9/10	9/10	9/10
HC <3rd percentile for age/sex	No	No	No	No	No	Yes	No	Yes	No	No	No
Admitted to NICU	No	No	No	No	No	No	No	Yes	No	No	No
Cranial ultrasound	negative	NT	NT	NT	negative	negative	NT	negative	NT	NT	NT
Age at follow-up (mos)	0,2,6,12	0,2,6,12	0	0,2,6,12	0	0,2,6,12	0,2,6,12	0,2,6,12	0,2,6,12	0,2,5,^††^12	0,2,6,12

**Abbreviations:** conf. = confirmed; HC = head circumference; IgM = immunoglobulin M; NICU = neonatal intensive care unit; NT = not tested; PCR = polymerase chain reaction testing; PRNT = plaque reduction neutralization test; prob. = probable.

* At birth and ages 2, 6, and 12 months

^†^ Zika-specific IgM antibodies and Zika virus-specific neutralizing antibodies in the same or a later specimen. Neutralizing antibodies detected by PRNT.

^§^ Two mothers had serum collected within 2 weeks of illness onset for PCR testing (number 6 at day 1 and number 8 at day 12). The remaining nine mothers (four symptomatic and five asymptomatic) had serum collected within 2 weeks of returning from an area with endemic Zika virus transmission for PCR testing.

^¶^ Dengue-specific IgM antibodies and dengue virus-specific neutralizing antibodies in the same or a later specimen. Neutralizing antibodies detected by PRNT.

** Specimens for serum and urine testing were collected within two days of birth.

^††^ Infant was evaluated at age 5 months, because the mother was unavailable for visit at age 6 months.

Although no infant had clinical or laboratory evidence of Zika virus infection, there were instances when laboratory or clinical information raised concern for possible Zika-associated birth defects. Zika virus RNA was isolated from the umbilical cord at the time of delivery of infant number 1 (Table); this infant had a negative Zika IgM test and was found to be healthy and normal at birth and at all follow-up visits. A fetal cranial ultrasound obtained for infant number 4 indicated “poor fetal brain development”; however, the mother’s amniotic fluid tested negative for Zika virus RNA, the infant tested negative for Zika virus at birth (serum IgM and RNA and urine RNA), and was healthy and normal at all follow-up visits. 

The head circumferences at birth of infant number 6 (30 cm) and infant number 8 (31 cm) were below the third percentile for gestational age and sex. Zika virus test results (serum IgM and RNA and urine RNA) were all negative for infant number 6 at birth; the infant received a diagnosis of microcephaly at age 1 week, but head circumference was normal at ages 2, 6, and 12 months, and a cranial ultrasound at age 3 months was unremarkable. A pediatrician classified the infant as normal at age 12 months. Infant number 8 was born at 38 weeks gestation, weighing 2.2 kg. Zika virus test results (serum IgM and RNA and urine RNA) at birth were negative. The infant received a diagnosis of symmetric growth retardation and was admitted to the neonatal intensive care unit for respiratory distress but was discharged home in good health at age 4 days. A pediatrician found this infant to be healthy and with normal head circumference at age 12 months.

Among 11 infants born to women in LAC with evidence of confirmed or probable Zika virus infection during pregnancy, the nine who participated in follow-up through age 12 months had no apparent adverse health effects at that time. Subtler health effects, or health effects occurring later in life, would not be captured with this surveillance activity. In addition, mothers with Zika virus infection who did not seek medical care, as well as those who chose not to participate in, or did not complete, the surveillance, limited the generalizability of these findings. Ongoing assessment of the health of infants born to women with evidence of Zika virus infection during pregnancy is important to assess the public health impact of Zika virus and to guide interventions.

## References

[1] de Araújo TVB, Rodrigues LC, de Alencar Ximenes RA, ; Investigators from the Microcephaly Epidemic Research Group; Brazilian Ministry of Health; Pan American Health Organization; Instituto de Medicina Integral Professor Fernando Figueira; State Health Department of Pernambuco. Association between Zika virus infection and microcephaly in Brazil, January to May, 2016: preliminary report of a case-control study. Lancet Infect Dis 2016;16:1356–63. 10.1016/S1473-3099(16)30318-8 27641777 PMC7617035

[2] Rasmussen SA, Jamieson DJ, Honein MA, Petersen LR. Zika virus and birth defects—reviewing the evidence for causality. N Engl J Med 2016;374:1981–7. 10.1056/NEJMsr1604338 27074377

[3] Kleber de Oliveira W, Cortez-Escalante J, De Oliveira WT, Increase in reported prevalence of microcephaly in infants born to women living in areas with confirmed Zika virus transmission during the first trimester of pregnancy—Brazil, 2015. MMWR Morb Mortal Wkly Rep 2016;65:242–7. 10.15585/mmwr.mm6509e2 26963593

[4] Cauchemez S, Besnard M, Bompard P, Association between Zika virus and microcephaly in French Polynesia, 2013–15: a retrospective study. Lancet 2016;387:2125–32. 10.1016/S0140-6736(16)00651-6 26993883 PMC4909533

[5] Adebanjo T, Godfred-Cato S, Viens L, ; Contributors. Update: interim guidance for the diagnosis, evaluation, and management of infants with possible congenital Zika virus infection—United States, October 2017. MMWR Morb Mortal Wkly Rep 2017;66:1089–99. 10.15585/mmwr.mm6641a1 29049277 PMC5689094

[6] CDC. Zika virus 2016 case definitions. Atlanta, GA: US Department of Health and Human Services, CDC; 2017. https://wwwn.cdc.gov/nndss/conditions/zika

